# Drug Allergy and the Risk of Lymph Node Metastasis in Rectal Cancer

**DOI:** 10.1371/journal.pone.0106123

**Published:** 2014-08-27

**Authors:** Chun Gao, Jing-Tao Li, Long Fang, Ying-Ying Xu, Hong-Chuan Zhao

**Affiliations:** Department of Gastroenterology, China-Japan Friendship Hospital, Ministry of Health, Beijing, P. R. China; University of Campinas, Brazil

## Abstract

**Background:**

Previous epidemiologic studies have reported that a history of allergy is associated with reduced risk of colorectal cancer and other malignancies. However, no information is available for the association between allergy and the risk of lymph node metastasis. Our study was designed to determine this association in rectal cancer.

**Methods:**

Patients who were treated at our hospital in the period from January 2003 to June 2011, and with a pathologically hospital discharge diagnosis of rectal adencarcinoma, were included. The clinical, laboratory, and pathologic parameters were analyzed. A multivariate logistic regression model was used to determine the association. Moreover, for type of allergic drug, sub-group analysis was performed.

**Results:**

469 patients were included, including 231 with pathological lymph node metastasis (pLNM) (49.3%) and 238 without pLNM. Univariate analysis showed, compared with patients without pLNM, patients with pLNM had a younger age (60.6±12.8 yr *vs.* 63.6±12.2 yr, P = 0.012), a lower percentage of drug allergy (8.7% *vs.* 16.0%, P = 0.016), an increased CEA (median/interquartile-range 5.40/2.40–13.95 *vs.* 3.50/2.08–8.67, P = 0.009), and a lower serum sodium (141±3.1 mmol/L vs. 142±2.9 mmol/L, P = 0.028). Multivariate analysis showed that drug allergy was associated with a reduced risk of pLNM (OR = 0.553; 95% CI, 0.308–0.994; P = 0.048). In addition, our results showed that: (1) for tumor classification, patients with drug allergy had a higher percentage of group patients with pT1/pT2; and (2) for type of allergic drug, this inverse association was found for penicillins, not for other allergic drugs.

**Conclusion:**

Drug allergy is associated with a reduced risk of pLNM in rectal cancer.

## Introduction

Rectal cancer (RC) is one of the most common malignant diseases worldwide and the prognosis is still poor although much progress has been achieved in recent years [1], [2]. Lymph node metastasis (LNM) is an important indicator of oncologic outcome for RC patients [3]. Previous studies have demonstrated that the number of retrieved lymph nodes is significantly associated with relapse and survival rates [4], [5]. The inability to examine a sufficient number of lymph nodes may lead to failure in identifying metastatic lymph nodes, and thus portend a worse prognosis [6]. Some clinicopathologic factors have been found to be associated with LNM in rectal cancer, including age<60 years, tumor diameter, tumor location, depth of invasion, poor differentiation, lymphovascular invasion and perineural invasion [7]–[11].

Drug allergy/hypersensitivity is a common problem seen by general and subspecialty adult and pediatric outpatient clinics, inpatient wards, and emergency department, which comprises about 10% to 30% of all adverse drug reactions [12]. Drug allergy develops through immunologic mechanisms and has different clinical manifestations, the most common of which is skin damage, which manifests through type 1 and type 4 hypersensitivity reactions [12]. An inverse association between allergy and cancer has been suspected for a long time, but even despite extensive research no general relationship has been determined [13], [14]. A review, which analyzed epidemiologic literature since 1985, concluded that atopy (immunoglobulin E-mediated allergy) is associated with decreased overall cancer risk with consistent findings for childhood leukemia and brain and pancreatic cancers [15].

Several case-control studies have suggested that allergy might play a protective role in the carcinogenesis of the rectum [16]–[19]; however, data reported by cohort studies are inconsistent [20]–[22]. The largest prospective study published to date, the Cancer Prevention Study II, which included a prospective cohort study of 1,102,247 US men and women who were cancer-free at baseline, reported an inverse association between colorectal cancer mortality and a history of both asthma and hay fever in comparison with persons with neither of these allergic conditions [23]. However, for the association between drug allergy and risk of LNM, no information is available. Our study was designed to determine this association in rectal cancer.

## Patients and Methods

### Study population

The patients who were treated at our hospital in the period from January 2003 to June 2011, and with a pathologically hospital discharge diagnosis of rectal adencarcinoma, according to the diagnostic, inclusion and exclusion criteria, would be included in our study. Patients following these criteria would be excluded: 1) those who had been treated by any method at inclusion or with pathological diagnosis for more than 15 days; 2) those who did not have a whole pathological data, including palliative operation and those whose operation were performed at other hospitals; 3) those who survived for less than three months after surgery; 4) those who had a presence of other malignancies, including lymphoma and leukemia; 5) those who had been treated by cytotoxic drugs and immunosuppressive agents in the past six months; 6) those who were diagnosed with hereditary non-polyposis colorectal cancer, inflammatory bowel disease and familial adenomatous polyposis; and 7) those who had a presence of serious disease of other important organs or systems, and rheumatic diseases.

The study was approved by the Hospital Human Research Ethics Committee of our hospital (Ethics Committee for Clinical Trials of Drugs and Devices of China-Japan Friendship) and it was in accordance with the principles of the *Declaration of Helsinki*. The patient records/information was anonymized and de-identified prior to analysis as the written informed consent was not obtained.

### Determination of rectal cancer, lymph node metastasis and drug allergy

The diagnosis of rectal cancer was histologically confirmed by the biopsy or surgical specimens. Only those patients with adencarcinoma were included in our study. When two and more histological types were found in the same patient and adencarcinoma was the major type, they would also be excluded from our analysis. Lymph node metastasis was diagnosed based on the histological findings of surgery, and they were named as pathological lymph node metastasis (pLNM). Based on the operative notes, the grade of pathological lymph node stage (pN), pathological tumor classification (pT) and pathological distant metastasis (pM) were determined. Drug allergy was preliminarily determined according to the self-reported results in the inpatient medical records. The contact details of patients with allergy history were recorded, and then we contacted with these patients and confirmed the information about drug allergy. When different results were found, the final was determined based on the information of further contact.

### Clinical, laboratory and pathological parameters

According to the inpatient medical records and our current knowledge, these clinical, laboratory and pathological parameters were included in our study, including gender, age, body mass index (BMI), drug allergy, hypertension, alcohol intake, smoking, white blood cell, hemoglobin, platelet count, total bilirubin, alanine aminotransferase (ALT), albumin level, creatinine, serum sodium, potassium, carcinoembryonic antigen (CEA), CA19-9, tumor differentiation, pT, pN and pM.

### Follow-Up

Patients were followed after surgery by serial clinical examination and CEA assessment every 3 months during the first year, every 6 months during the second year, and annually thereafter. Thoracoabdominal computed tomography (CT) scanning was performed every 6 months for the first 2 years. Colonoscopy was performed after 1 year and 3 to 5 years thereafter, depending on individual patient risk. Further diagnostic methods were used as required if recurrence was suspected.

### Statistical analysis

The Statistical Package for the Social Sciences (SPSS version 19.0; Chicago, Ill, USA) was used for data management and statistical analyses. For the continuous variables, mean±standard deviation was described and Independent-Samples T-test was used. If the continuous variable has skewed distribution, it would be described as the median and inter-quartile range and analyzed by Mann–Whitney non-parametric U-test. For categorical variables, the numbers and proportions of patients in each group were described, and Pearson Chi-Square test, continuity correction Chi-Square tests or Fisher’s exact test were used. Based on the results of univariate analysis, multivariate unconditional logistic regression model was used to determine the association between drug allergy and the risk of lymph node metastasis. Stepwise multiple regression analysis (Backward: Wald; Entry: 0.05, Removal: 0.10) was used. We expressed the results as odds ratios (ORs) and their 95% confidence intervals (CIs).

Moreover, we would study the association between clinicopathological data and drug allergy, and the association between type of allergic drug and lymph node metastasis. For prognostic significance, the Kaplan-Meier method (log-rank test) and multivariate Cox regression model were used to determine the association of pLNM and some parameters with survival time. The stability of the Cox model was tested by bootstrap resampling, which was a useful procedure to test the internal stability of a model proposed [24], [25]. It consists of creating new data sets of equal size by random sampling of the original data with replacement. In an individual new bootstrap sample, a patient may be represented once, multiple times or not at all. A new Cox regression was then calculated for each of these new data sets in order to obtain the bootstrap parameter estimates. We expressed the results of Cox model as hazard rates (HRs) and their 95% confidence intervals (CIs). P value was used for the results of bootstrap resampling. For all tests, P<0.05 was considered statistically significant and all P values quoted are two-sided.

## Results

### Study population and basic characteristics

A total of 469 patients diagnosed with rectal adenocarcinoma were included in our study, including 231 patients with pathological lymph node metastasis (pLNM) (49.3%) and 238 patients without pLNM. The basic characteristics were demonstrated in **Table 1**. Sixty-one percent (286/469) were male and the mean age was 62.1±12.6 years old. Fifty-eight patients (12.4%) had allergic history and 129 (27.5%) were smoking. Pathological characteristics were shown in **Table 2**, including tumor differentiation, pathological tumor classification (pT), pathological lymph node stage (pN) and pathological distant metastasis (pM). In the 231 patients with pLNM, 136 (136/231 = 58.9%) were diagnosed with lymph node stage 1 (pN1).

**Table 1:** Basic characteristics of 469 patients with rectal adenocarcinoma (RAC).

Characteristic	Total patients(n = 469)*	RAC patients with LNM(n = 231)*	RAC patients without LNM(n = 238)*	P Value
**Gender, male (n, %)**	286 (61.0)	146 (63.2)	140 (58.8)	0.331
**Age (yr)**	62.1±12.6	60.6±12.8	63.6±12.2	**0.012**
**BMI^a^ (kg/m^2^)**	23.95±3.54	23.90±3.67	24.0±3.41	0.779
**Drug allergy (n, %)**	58 (12.4)	20 (8.7)	38 (16.0)	**0.016**
**Hypertension (n, %)**	131 (27.9)	58 (25.1)	73 (30.7)	0.179
**Alcohol intake (n, %)**	29 (6.2)	18 (7.8)	11 (4.6)	0.154
**Smoking (n, %)**	129 (27.5)	55 (23.8)	74 (31.1)	0.077
**White blood cell (×9/L)**	6.66±2.10	6.70±2.09	6.61±2.11	0.666
**Hemoglobin (g/L)**	132±20	131±20	132±19	0.553
**Platelet count (×9/L)**	233±79	235±74	232±84	0.659
**Total bilirubin** † **(µmol/L)**	10.26 (7.70–14.40)	10.26 (7.40–15.0)	10.26 (8.13–13.68)	0.451
**ALT** † **(U/L)**	16 (12–22)	15 (12–22)	16 (12–22)	0.262
**Albumin level^b^ (g/L)**	42.3±4.5	42.3±4.4	42.2±4.6	0.908
**Creatinine (mg/dL)**	82.1±18.0	80.8±15.5	83.3±20.1	0.128
**Serum sodium^c^ (mmol/L)**	141±3.0	141±3.1	142±2.9	**0.028**
**Serum potassium^c^ (mmol/L)**	4.1±0.5	4.1±0.4	4.1±0.5	0.282
**Carcinoembryonic antigen^d^** † **(ng/ml)**	4.33 (2.22–11.91)	5.40 (2.40–13.95)	3.50 (2.08–8.67)	**0.009**
**Serum CA19-9^e^** † **(kU/L)**	14.02 (7.20–28.63)	15.10 (7.39–33.91)	13.41 (7.10–23.10)	0.109

BMI, body mass index; LNM, lymph node metastasis; RAC, rectal adenocarcinoma.

Data were available in^ a^403 (202+201), ^b^463 (227+236), ^c^457 (226+231), ^d^392 (192+200) and ^e^370 (178+192) patients. The numbers before the brackets indicate the total available cases in the two groups.

*Plus-minus value indicates mean±standard deviation;

† Median (inter-quartile range, Q1– Q3).

**Table 2:** Pathological characteristics and follow-up results of 469 RAC patients.

Characteristic	Total patients(n = 469)*	RAC patients with LNM(n = 231)*	RAC patients without LNM(n = 238)*	P Value
**Tumor differentiation**				
** Moderately/well (n, %)**	388 (82.7)	166 (71.9)	222 (93.3)	**<0.001**
** Poorly (n, %)**	57 (12.2)	46 (19.9)	11 (4.6)	–
** Unknown (n, %)**	24 (5.1)	19 (8.2)	5 (2.1)	–
**Pathological tumor classification (pT)**				**<0.001**
** pT1 (n, %)**	18 (3.8)	1 (0.4)	17 (7.1)	–
** pT2 (n, %)**	90 (19.2)	21 (9.1)	69 (29.0)	–
** pT3 (n, %)**	118 (25.2)	54 (23.4)	64 (26.9)	–
** pT4 (n, %)**	243 (51.8)	155 (67.1)	88 (37.0)	–
**Pathological lymph node stage (pN)**				**<0.001**
** pN0 (n, %)**	238 (50.7)	0 (0.0)	238 (100.0)	–
** pN1 (n, %)**	136 (29.0)	136 (58.9)	0 (0.0)	–
** pN2 (n, %)**	95 (20.3)	95 (41.1)	0 (0.0)	–
**Pathological distant metastasis (pM)**				
** pM0 (n, %)**	439 (93.6)	213 (92.2)	226 (95.0)	0.224
** pM1 (n, %)**	30 (6.4)	18 (7.8)	12 (5.0)	–
**Results of follow-up**				
** Death (n, %)**	72 (15.4)	46 (19.9)	26 (10.9)	**0.007**
** Survival (n, %)**	277 (59.1)	118 (51.1)	159 (66.8)	**0.001**
** Lost (n, %)**	120 (25.6)	67 (29.0)	53 (22.3)	0.095
** Time of follow-up** † **(months)**	24.0 (9.0–44.3)	20.0 (9.0–37.9)	29.1 (10.5–48.5)	**0.013**

† Median (inter-quartile range, Q1–Q3).

### Univariate analysis: comparison of rectal cancer patients with and without lymph node metastasis

In the 231 patients with pLNM, the mean age was 60.6±12.8 years and 146 (63.2%) patients were male. In the 238 patients without pLNM, the mean age was 63.6±12.2 years and 140 (58.8%) patients were male. As shown in **Table 1**, compared with patients without pLNM, the patients with pLNM had a younger age (60.6±12.8 yr *vs.* 63.6±12.2 yr, P = 0.012), a lower percentage of drug allergy (8.7% *vs.* 16.0%, P = 0.016), an increased level of CEA (median/interquartile-range 5.40/2.40–13.95 *vs.* 3.50/2.08–8.67, P = 0.009), and a lower value of serum sodium (141±3.1 mmol/L vs. 142±2.9 mmol/L, P = 0.028). Among the total, data were available in 457 patients for serum sodium and 392 patients for CEA. For pathological characteristics (**Table 2**), univariate analysis showed that between rectal cancer patients with and without pLNM, statistical differences were found for tumor differentiation (P<0.001), pathological tumor classification (pT) (P<0.001), not for distant metastasis (P = 0.454).

### Drug allergy and type of allergic drug

**Figure 1:**
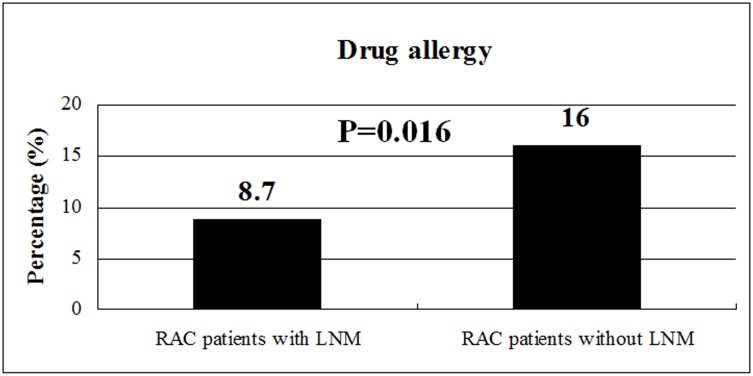
Univariate analysis showed that the percentage of drug allergy was lower in rectal adenocarcinoma (RAC) patients with pathological lymph node metastasis (pLNM) compared with those patients without pLNM (8.7% vs. 16.0%, P = 0.016).

**Figure 2:**
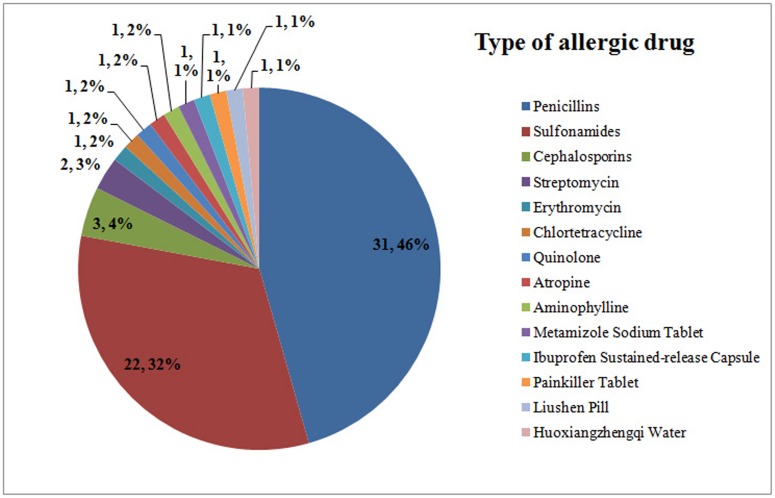
Fifty-eight patients had drug allergy, including 50 patients having allergic reactions to one kind of drug, six patients to two kinds of drugs and two patients to three kinds of drugs. The two numbers indicate the number and percentage of patients.

Among the 469 patients, 58 (12.4%) had drug allergy, including 20 in the group patients with pLNM and 38 patients in another group without pLNM. Univariate analysis showed that the percentage of drug allergy was lower in patients with pLNM compared with those patients without pLNM (8.7% *vs.* 16.0%, P = 0.016) (**Figure 1**). For the number of allergic drug, 50 patients had allergic reactions to one kind of drug, six patients to two kinds of drugs and two patients to three kinds of drugs. The type of allergic drug was shown in **Figure 2**. Thirty-one (31/58 = 53.4%) patients had allergic reactions to penicillins and 22 (22/58 = 37.9%) patients to sulfonamides, which accounted for almost eighty percent of drug allergy. Other allergic drugs included cephalosporins, streptomycin, erythromycin, chlortetracycline, quinolone, atropine, aminophylline, metamizole sodium tablet, ibuprofen sustained-release capsule, painkiller tablet, Liushen pill and Huoxiangzhengqi water (**Figure 2**).

### Multivariate analysis: drug allergy and the risk of lymph node metastasis

Multivariate unconditional logistic regression model was used to determine the association between drug allergy and the risk of pLNM. CEA was excluded from multivariate analysis because data were not available in 77 patients and preliminary results showed that CEA was not associated with LMN in our patients (data not shown). Four hundred and fifty-seven patients were included because the data were not available for 12 patients. When age, drug allergy and serum sodium were included in multivariate analysis (**Table 3**), drug allergy was found to be associated with the reduced risk of pLNM in RC (OR = 0.553; 95% CI, 0.308–0.994; P = 0.048).

**Table 3:** Multivariate analysis for drug allergy and the risk of lymph node metastasis*.

Variable	Adjusted OR	95% CI	P Value
**Age (yr)**	0.985	0.970–1.000	0.053
**Drug allergy**	0.553	0.308–0.994	**0.048**
**Serum sodium (mmol/L)**	0.940	0.882–1.002	0.057

CI: confidence intervals; OR: odds ratios.

*457 patients were included because the data were not available for 12 patients. Carcinoembryonic antigen (CEA) was excluded because those were not available in 77 patients and preliminary results showed that CEA was not associated with LMN by multivariate analysis in our patients.

### Association between drug allergy and clinicopathological data

According to the results from univariate analysis shown in **Table 1** **–** **2**, association between drug allergy and some clinicopathological data were studied using chi-square test, including gender, age, serum sodium, CEA, tumor differentiation, pT, pN and pM. Our results showed that (**Table 4**) drug allergy was associated with age (P = 0.016) and pathological tumor classification (P = 0.027). When patients were sub-grouped as those with pT1/pT2 or pT3/pT4, as shown in **Table 4**, patients with drug allergy had a higher percentage of group patients with pT1/pT2 (34.5% *vs.* 21.4%, P = 0.027). However, for the grade of pathological lymph node stage, tumor differentiation and pathological distant metastasis, no statistically significant differences were found.

**Table 4:** Association between drug allergy and clinicopathological data.

Variable	Patients with drug allergy		Patients without drug allergy		P
	Number	Percent	Number	Percent	
**Gender**					
** Male**	35	60.3	251	61.1	0.916
** Female**	23	39.7	160	38.9	–
**Age, year**					
** <60**	14	24.1	167	40.6	**0.016**
** ≥60**	44	75.9	244	59.4	–
**Serum sodium, mmol/L**					
** ≤141**	25	43.1	218	54.6	0.100
** >141**	33	56.9	181	45.4	–
**Carcinoembryonic antigen, ng/ml**					
** <5.0**	21	38.9	157	46.4	0.300
** ≥5.0**	33	61.1	181	53.6	–
**Tumor differentiation**					
** Moderately/well**	50	90.9	338	86.7	0.378
** Poorly**	5	9.1	52	13.1	–
**Pathological tumor classification (pT)**					
** pT1/pT2**	20	34.5	88	21.4	**0.027**
** pT3/pT4**	38	65.5	323	78.6	–
**Pathological lymph node stage (pN)**					
** pN1**	12	60.0	124	58.8	0.915
** pN2**	8	40.0	87	41.2	–
**Pathological distant metastasis (pM)**					
** pM0**	56	96.6	383	93.2	0.488
** pM1**	2	3.4	28	6.8	–

### Association between type of allergic drug and lymph node metastasis

Considering that fourteen kinds of drugs were included in our analysis, we would study the association between type of allergic drug and lymph node metastasis (**Table 5**). Those patients were sub-grouped as three groups according to the type of allergic drug and number of patients with allergic reactions, including penicillins group, sulfonamides group and other drugs group. Among the total, 31 patients had allergic reactions to penicillins, including 22 patients in group without pLNM and 9 patients in another group with pLNM. Statistical analysis (**Table 5**) showed that patients without pLNM had a higher percentage of patients with allergic reactions to penicillins (9.2% *vs.* 3.9%, P = 0.020). The OR was 0.398 and 95% CI was 0.179-0.884. For sulfonamides and other drugs, no statistically significant differences were found.

**Table 5:** Association between type of allergic drug and lymph node metastasis.

Type of drug	Patients with LNM*		Patients without LNM		P value	OR	95% CI
	Number	Percent	Number	Percent			
**Penicillins**							
** Allergy**	9	3.9	22	9.2	**0.020**	**0.398**	**0.179–0.884**
** Non-allergy**	222	96.1	216	90.8	–	–	–
**Sulfonamides**							
** Allergy**	9	3.9	13	5.5	0.423	0.702	0.294–1.675
** Non-allergy**	222	96.1	225	94.5	–	–	–
**Others**							
** Allergy**	6	2.6	7	2.9	0.821	0.880	0.291–2.659
** Non-allergy**	225	97.4	231	97.1	–	–	–

CI: confidence intervals; LNM: lymph node metastasis; OR: odds ratios.

*Of the 58 patients with drug allergy, six patients had allergic reactions to two kinds of drugs and two patients had reactions to three kinds of drugs. Their ORs and 95% CIs were calculated using Pearson Chi-Square tests.

### Follow-up and survival analysis

**Figure 3:**
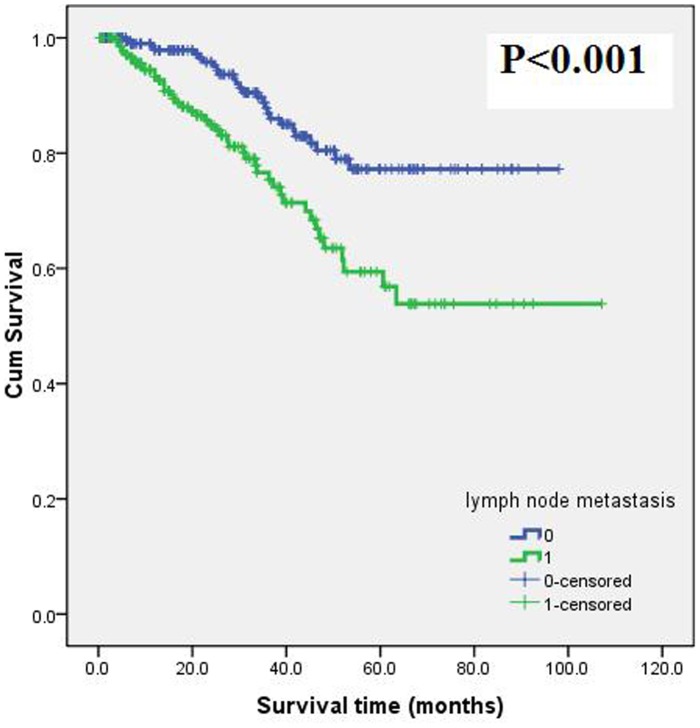
Survival analysis: Kaplan-Meier curve (log rank test) showed lymph node metastasis was associated with the prognosis (survival time).

The median follow-up was 24.0 months (range, 0.4–107.1 months; inter-quartile range, 9.0–44.3 months). One hundred and twenty patients (25.6%) were lost, including 67 in the group with pLNM and 53 in those without pLNM (P = 0.095). Seventy-two (15.4%) patients were dead, univariate analysis (**Table 2**) showed that the death was higher in patients with pLNM than in patients without pLNM (P = 0.007). The Kaplan-Meier curve and log rank test (**Figure 3**) showed that pLNM was associated with the prognosis (P<0.001), which was supported by the Cox regression analysis.

Combined the results of uni- and multivariate analysis with our current knowledge, five variables were included in Cox regression analysis, including age, gender, drug allergy, pLNM and serum sodium. The result showed that pLNM (HR = 2.892; 95% CI, 1.734–4.823; P<0.001) and age (HR = 1.023; 95% CI, 1.002–1.045; P = 0.036), not drug allergy (HR = 1.353; 95% CI, 0.682–2.683; P = 0.387), were associated with the prognosis. The stability of Cox model was tested by bootstrap resampling. Results of bootstrap, which were based on 1000 bootstrap samples, showed that pLNM was the only variable with statistical difference (P = 0.001) for prognosis of these patients.

## Discussion

Our results found that, compared with patients without pLNM, patients with pLNM had a younger age, a lower percentage of drug allergy (8.7% *vs.* 16.0%, P = 0.016), an increased CEA and a lower serum sodium. Multivariate analysis showed that drug allergy was associated with the reduced risk of pLNM in RC (OR = 0.536; 95% CI, 0.299–0.959; P = 0.036). In addition, we found that: (1) for the association between drug allergy and clinicopathological data, drug allergy was associated with an older age and a better pathological tumor classification. Patients with drug allergy had a higher percentage of group patients with pT1/pT2; and (2) for the association between type of allergic drug and pLNM, sub-group analysis showed that penicillins accounted for the major reason, not other allergic drugs.

For the first time, our study was designed to determine the association between drug allergy and the risk of lymph node metastasis in malignant diseases, let alone in rectal cancer. The association between allergic conditions or diseases and colorectal cancer has been reported by some previous epidemiologic studies [16]–[22]. Case-control studies have provided evidences that allergy may be associated with the reduced risk in the carcinogenesis of the colon and rectum [16]–[19]; however, data reported by cohort studies are inconsistent [20]–[22]. Fortunately, the largest prospective study published to date, the Cancer Prevention Study II, showed that allergic conditions (asthma and hay fever) was associated with a significantly lowered risk of colorectal cancer mortality [23]. However, as shown in these above-mentioned studies, almost all of them were designed for colorectal cancer, not simply for rectal cancer.

The association between allergy and other cancer development has also been well documented by a number of epidemiologic studies; however, the existence of such an association remains controversial. Inverse associations have been reported in case- control and cohort studies of glioma [26], [27], meningioma [28], [29], non-Hodgkin’s lymphoma [30], leukemia [31], and pancreatic cancer [32]. However, a history of asthma has been positively associated with lung cancer [33], and results for all cancers combined are unclear [34], [35]. Some studies have suggested that persons with a history of allergy may demonstrate enhanced immune function [23].

The major concern in our study was that the determination of drug allergy was mostly based on self-reporting of allergic conditions, which may raise the possibility that association may be non-causal and arise as a consequence of bias, reverse causation or other artifacts [26]. Some studies have used questionnaires to measure allergic history and showed that questions on self-reported physician-diagnosed allergic conditions were highly specific and more reliable than symptom-based questions or questionnaires asking respondents if they have ever had the disease [23]. However, our study was limited to the drug allergy and other allergic conditions or diseases had been excluded, which has reduced the bias to a lowest degree. Moreover, these drug allergies were preliminarily determined based on self-reported results in the inpatient medical records which had been diagnosed by the physicians. In addition, we had contacted with these patients by the contact details and confirmed their information.

The second concern was the nature of our hospital-based case-control study which raised the possibility of selection bias and limited control for confounding. However, our study was designed to determine the association between drug allergy and lymph node metastasis in rectal cancer patients. The same rectal cancer patients, not healthy persons, were served as control, which have reduced the selection bias and control for possible associated confounding factors to the minimum degree. In addition, we provided some additional evidences for the association, for example: (1) the similar inverse association was demonstrated between drug allergy and pathological tumor classification, which obviously has a close relationship with pLNM; and (2) even when the sub-group analysis was limited to the association between pLNM and penicillins, which were used widely in clinical practice and almost everyone knows the consequence of allergic reactions, the result remained unchanged.

For the association between drug allergy and clinicopathological data, we found that drug allergy was associated with an older age, which was inversely associated with lymph node metastasis. These results were supported by some previously published studies [8], [36]. Kim JS et al. reported that age<60 yr was an independent risk factor of proximal lymph node involvement (PLNp) in patients with node positive rectal cancer. Patients with PLNp had poorer oncologic outcomes than those without PLNp in terms of overall survival [36]. This conclusion was supported by another study, which was performed by Ding PR et al. to identify risk factors of LNM in T2 rectal cancer, showed that age was an independent predictor for overall LNM [8]. Studies on other carcinomas, such as breast cancer, also reported the similar results [37], [38].

Considering that fourteen kinds of drugs were included in our study and some of them may not be used by most of the patients, sub-group analysis was performed. Based on the type of allergic drug and number of patients with allergic reactions, the patients were sub-grouped as three groups, including penicillins group, sulfonamides group and other drugs group. Penicillins were used widely in clinical practice and almost everyone knows whether he or she was allergic to penicillin. When the analysis was limited to penicillins, a similar result was gained, which provide supporting evidence.

Two contradictory theories have been proposed: the theory of immune surveillance suggests that allergic conditions could reduce cancer risk by enhancing the ability of the immune system to detect and remove malignant cells, whereas another theory argues that allergy is accompanied by repeated tissue inflammation, damage, and repair, which increases the risk of cancer [16]. This observed inverse association, if causal, may reflect enhanced immunosurveillance in allergic participants (i.e., the enhanced ability of immune system to detect and eliminate cancer cells before they become clinically manifest), which is supported by experimental studies that show that allergy is accompanied by immunoglobulin E production, a significant decrease in tumor occurrence and growth, and an increase in survival time [16], [39].

In conclusion, we found that drug allergy is associated with a reduced risk of pLNM in rectal cancer, although more studies are required for a better understanding.
